# A geospatial database of drought occurrence in inland valleys in Mali, Burkina Faso and Nigeria

**DOI:** 10.1016/j.dib.2018.06.105

**Published:** 2018-06-30

**Authors:** Elliott R. Dossou-Yovo, Amadou M. Kouyaté, Tasséré Sawadogo, Ibrahima Ouédraogo, Oladele S. Bakare, Sander J. Zwart

**Affiliations:** a Africa Rice Center (AfricaRice), BFJ BFJ613609; b Institut d׳Economie Rurale (IER), Sikasso, Mali; c Ministère de l’Agriculture et de la Sécurité Alimentaire (MASA), Ouagadougou, Burkina Faso; d Institut de l׳Environnement et Recherches Agricoles (INERA), Bobo Dioulasso, Burkina Faso; e National Cereals Research Institute (NCRI), Bida, Niger State, Nigeria; f Faculty of Geo-Information Science and Earth Observation (ITC), University of Twente, Netherlands

**Keywords:** Drought, Inland valleys, Rice, Africa

## Abstract

The data described in this article are related to drought occurrence in inland valleys and farmers adaptation strategies. The data were collected in 300 inland valleys distributed in 14 regions of West Africa. The data were collected in two phases. In the first phase, 300 inland valleys were identified in 14 regions and their locations were determined with handheld GPS devices. Questionnaires and informal interviews were administered to inland valleys users to collect data on physical and socio-economic characteristics, hydrology, farmers experience with drought affecting rice production in inland valleys and adaptation strategies. In the second phase, the locations of the inland valleys were imported in a GIS environment and were used to extract additional parameters on soil characteristics and water demand from the Shuttle Radar Topography Mission (SRTM), Africa Soil Information Service (africasoils.net) and POWER database (http://power.larc.nasa.gov). In total, the dataset contains 41 variables divided into seven themes: farmers’ experience with drought, adaptive management of rice farmers to drought, physical characteristics, hydrology, management practices, socio-economic characteristics and weather data of inland valleys.

**Specifications Table**

**Table:** 

Subject area	Environmental Sciences, Social Sciences
More specific subject area	Climate, Food security, Agriculture
Type of data	Table (Excel format)
How data were acquired	Face-to-face farmer surveys using questionnaires and informal interviews, geographic locations obtained with handheld GPS devices, secondary data extracted from maps using geographic coordinates (polygon shape files).
Data format	Raw, cleaned
Experimental factors	Not applicable
Experimental features	Not applicable
Data source location	The data were collected in 14 administrative zones in 3 countries, see also Fig. 1.
	Mali, 1 region:
	1. Sikasso
	Nigeria, 2 states:
	2. Niger state
	3. Kaduna state
	Burkina Faso, 11 regions
	4. Boucle du Mouhoun
	5. Cascades
	6. Centre
	7. Centre Est
	8. Centre Nord
	9. Centre Ouest
	10. Centre Sud
	11. Est
	12. Hauts Bassins
	13. Plateau Central
	14. Sud-Ouest (Burkina Faso)
	The geographic coordinates of each inland valley are included in the data base.
Data accessibility	Data are provided with this article

**Value of the data**

• Large multidisciplinary dataset comprising 300 inland valleys in 14 regions distributed in 3 countries in West-Africa, covering location, physical characteristics, socioeconomic characteristics, hydrology, weather data, farmers management practices, farmers experience with drought affecting rice production in inland valleys and adaptation strategies.
• The dataset can be deployed to assess the impacts of drought on rice production, to classify farmers management approaches to mitigate drought in inland valleys, to characterize the diversity of inland valleys based on biophysical and socio-economic characteristics, to analyze suitability of inland valleys for rice-based production systems, etc.
• The data can be linked to similar surveys conducted in Benin, Liberia and Sierra Leone [1], [2], [3] to analyze the determinants of farmers decision-making with respect to agricultural use of inland valleys in West Africa.
• The dataset contributes to spatial assessment of agricultural drought and to food security research in West Africa.

## 1 Data

Inland valley ecosystems are estimated to cover 190 Mha in Africa. Inland valleys are defined as the upper parts of river drainage systems, comprising the whole upland lowland continuum, from the rainfed uplands (pluvial) to rainfed, flooded and intensified lowlands in the valley bottom (fluxial), with the hydromorphic fringes (phreatic) as the (sloping) transition zone between them [4]. Given the high agricultural production potential, inland valleys provide opportunities to improve food and nutrition security for smallholder farmer families in sub-Saharan Africa. Besides agricultural production, inland valleys provide local communities with forest, forage, hunting and fishing resources and recreational sites [1].

**Fig. 1:**
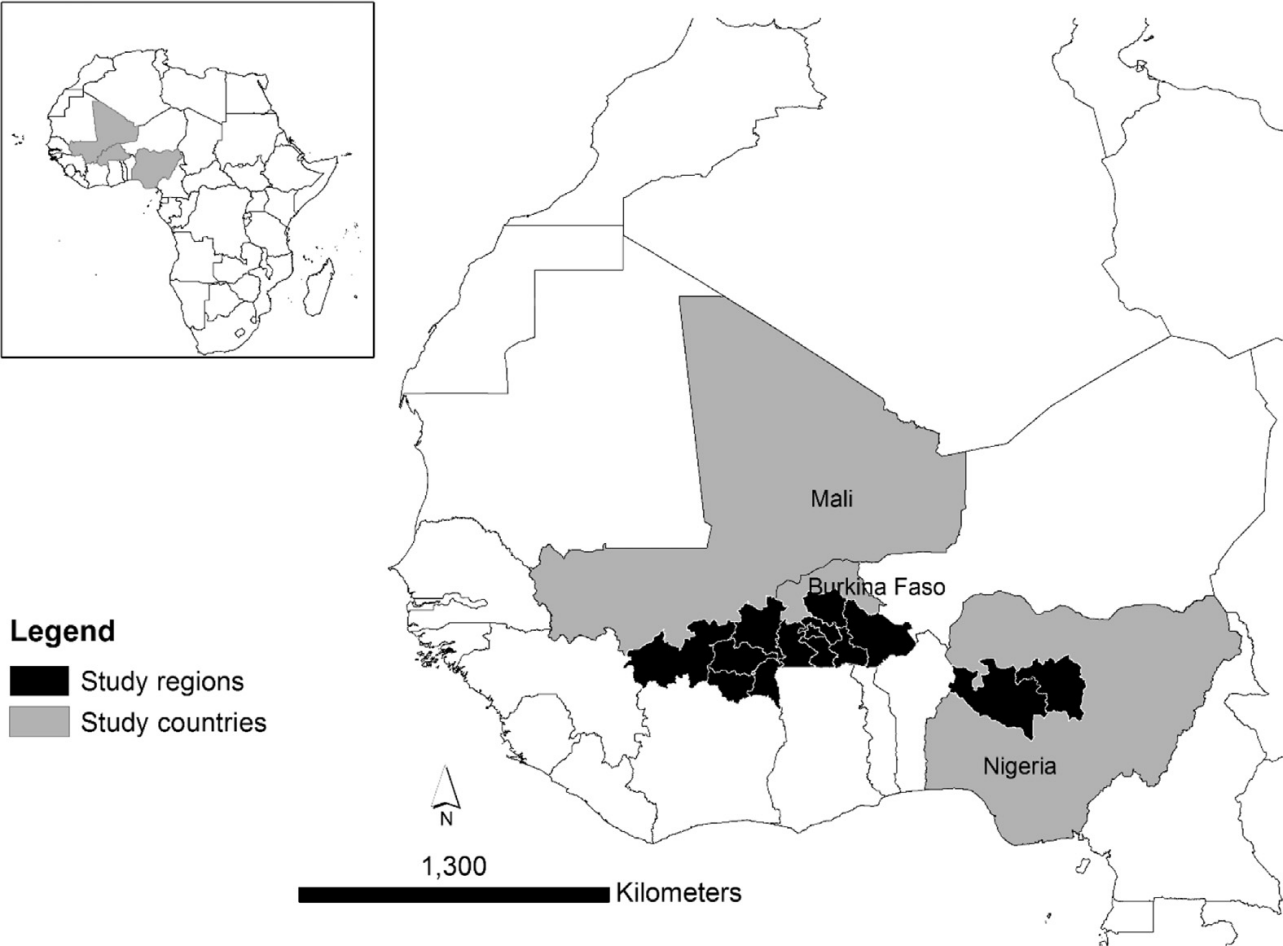
Location of the study area in West Africa.

The database contains physical, hydrological, socioeconomic and weather data, as well as farmers experience of drought and adaptation strategies. The data were collected in 300 inland valleys distributed in 14 regions of three West African countries: Mali (98 inland valleys), Nigeria (106) and Burkina Faso (96) (see Fig. 1). The 14 regions are located in the Sudan-Sahel zone where average annual rainfall varies from 700 to 1300 mm. The inland valleys are geolocated with latitude/longitude coordinates. For each inland valley, 41 variables, grouped in seven themes (Table 1), were obtained from either farmers’ responses during community surveys in inland valleys conducted in 2013 or from digital maps using the location (polygon shape file) of the inland valleys. Table 1 provides a summary of the data base and the included variables.

**Table 1:** Summary of the variables included in the inland valley database grouped by theme.

Variables	Scale type	Scale class	Source of data
Theme 1: Farmers’ experience with drought in the last 10 years			
Occurrence of drought	Nominal	Yes, no	Survey
Frequency of drought events	Ordinal	Every year, every 2 or 3 years, every 4 or 5 years, more than every 5 years, never	Survey
Frequency of entire rice harvest loss	Ordinal	All years, in 1 to 2 years, in 3 to 6 years, in 7 to 9 years, never	Survey
Frequency of rice yield reduction	Ordinal	All years, in 1 to 2 years, in 3 to 6 years, in 7 to 9 years, never	Survey

Theme 2: Adaptive management of rice farmers to drought			
Use of drought resistant varieties	Nominal	Yes, no	Survey
Change in cultivation areas	Nominal	Yes, no	Survey
Investment in irrigation facilities	Nominal	Yes, no	Survey
Change in cropping seasons	Nominal	Yes, no	Survey
Others	Nominal	Bund, bund + compost + mulching, bund + early sowing, bund+ early sowing+ organic fertilizer, bund+ organic manure, bund+ organic manure+ early sowing, bund+ organic manure+ irrigation, dry sowing+ organic manure, early sowing, irrigation, irrigation+ contour tillage, none, off-season cropping+ irrigation, organic manure, tillage+ organic manure, tree plantation, water conservation measures	Survey

Theme 3: Physical characteristics			
Inland valley size (ha)	Numeric	–	Digital elevation map
Average width (m)	Numeric	–	Digital elevation map
Cross-sectional shape	Nominal	Convex, concave, flat	Survey
Particle size distribution (%)	Numeric	–	AfSIS^a^
Soil organic carbon (%)	Numeric	–	AfSIS

Theme 4: Hydrology			
Water source	Nominal	Spring, river, other	Survey
Flooding regime	Ordinal	Sporadic, seasonal, permanent	Survey
Duration of flooding (week)	Numeric	–	Survey
Duration of emerging water table (week)	Numeric	–	Survey
Duration of shallow water table (week)	Numeric	–	Survey
Drainage/irrigation infrastructure	Nominal	No drainage, canals for drainage and/or irrigation	SRTM^b^
Flow accumulation	Numeric	–	SRTM

Theme 5: Management practices			
Rice varieties	Nominal	Only local, only improved, both local and improved	Survey
Soil fertility management	Nominal	No fertilizer, only mineral fertilizer, both mineral and organic fertilizers	Survey
Bunds	Nominal	No bunding, simple bunding, contour bunds	Survey

Theme 6: Socio-economic characteristics			
Distance to road and distance to market (km)	Numeric	–	Survey
Quality of road to market	Nominal	No road, path, dirt road, paved road	Survey
Land ownership	Nominal	Individual, family, village, state	Survey
Origin of inland valley users	Nominal	Native, migrant	Survey
Percentage of women in the inland valleys (%)	Numeric	–	Survey
Mode of exploitation	Nominal	Individual, collective, both	Survey
Source of seeds and other agricultural inputs	Ordinal	In the village, at < 25 km, 25–50 km, 51–100 km, > 100 km	Survey
Support from institution	Nominal	Yes, no	Survey
Affiliation with farmers’ organization	Nominal	Yes, no	Survey
Role of rice farming in production system	Nominal	Main activity, secondary major activity, marginal activity	Survey

Theme 7: Weather data			
Daily minimum temperature	Numeric	–	POWER database
Daily maximum temperature	Numeric	–	POWER database
Daily rainfall	Numeric	–	POWER database

a Africa Soil Information Service (AfSIS)

b Shuttle Radar Topography Mission (SRTM), URL: http://srtm.csi.org.

The data base is in Microsoft Excel format and contains eight sheets. The first sheet (variable explanation) provides an explanation of the variables. The second sheet (location) provides the unique identifier of each surveyed inland valley and the geographic coordinates expressed in longitude/latitude. The unique identifier can be linked to the variables stored in three sheets, one for each of the three countries, called *Mali*, *Nigeria* and *Burkina* Faso. The sheets *Mali-weather data*, *Nigeria-weather data and Burkina Faso-weather data* provide daily rainfall and minimum and maximum air temperatures from 1995 to 2014 for each surveyed inland valley.

## 2 Experimental design, materials and methods

This section provides a summary of the approaches followed to develop the geospatial data base. We refer to Dossou-Yovo et al. [5] for a full description of the methodology that was followed. Data were collected in two phases. In the first phase, 300 inland valleys were identified in 14 regions distributed in three West African countries located in the Sudan-Sahel zone, viz. Burkina Faso, Mali and Nigeria. The location of each inland valley was determined with handheld GPS devices. Data on physical and socio-economic characteristics, hydrology, farmers experience with drought in rice-based production systems and adaptation strategies were collected from small groups of 5 to 20 farmers for each inland valley based on questionnaires and informal interviews. In the second phase, the geographic locations of the inland valleys were imported in a GIS environment and their quality was checked. Spatial information available in the public domain were downloaded and imported in GIS. These included soil parameters (particle size distribution and soil organic carbon), flow accumulation, daily rainfall and minimum and maximum air temperatures data. Digital elevation data from the Shuttle Radar Topography Mission (SRTM) at a spatial resolution of 30 m were used to derive flow accumulation. Maps of soil parameters in the first 30 cm of soil depth were obtained from the Africa Soil Information Service (AfSIS) project website (africasoils.net). Gridded daily rainfall and temperature data for the period 1995–2014 were obtained from the POWER database (http://power.larc.nasa.gov/). Table 2 provides an overview of the 41 variables in the data base and their source (whether from the field surveys or public domain sources).
