# Supplementary material for: A geospatial database of drought occurrence in inland valleys in Mali, Burkina Faso and Nigeria
Source: Data Brief. 2018 Jun 30;19:2008–14. doi: 10.1016/j.dib.2018.06.105 (PMC6141420; doi:10.1016/j.dib.2018.06.105)
Supplement: Supplementary file 1 — Transparency document [file mmc1.pdf]

# CONFLICT OF INTEREST FORM

18 June 2018

Manuscript Title: A geospatial database of drought occurrence in inland valleys in Mali, Burkina Faso and Nigeria

Ms Number: DIB-D-18-01102

Authors: Elliott R. Dossou-Yovo, Amadou M. Kouyaté, Tasséré Sawadogo, Ibrahima Ouédraogo, Oladele S. Bakare, Sander J. Zwart

Corresponding Author: Dr. Elliott R. Dossou-Yovo

**The corresponding author confirms on behalf of all authors that the authors whose names are listed above have no affiliations with or involvement in any organization or entity with any financial interest (such as honoraria; educational grants; participation in speakers' bureaus; membership, employment, consultancies, stock ownership, or other equity interest; and expert testimony or patent-licensing arrangements), or non-financial interest (such as personal or professional relationships, affiliations, knowledge or beliefs) in the subject matter or materials discussed in this manuscript.**

Dr. Elliott R. Dossou-Yovo

Africa Rice Center (AfricaRice), Bouaké, Côte d'Ivoire
